# Investigating the role of Caspase-1 in a mouse model of Juvenile X-linked Retinoschisis

**DOI:** 10.3389/fmed.2024.1347599

**Published:** 2024-06-13

**Authors:** Ella J. Gehrke, Araniko Pandey, Jacob Thompson, Sajag Bhattarai, Prajwal Gurung, Ying Hsu, Arlene V. Drack

**Affiliations:** ^1^Department of Ophthalmology and Visual Sciences, IVR, University of Iowa, Iowa City, IA, United States; ^2^Department of Epidemiology, College of Public Health, University of Iowa, Iowa City, IA, United States; ^3^Division of Infectious Diseases, Department of Internal Medicine, University of Iowa Hospitals and Clinics, Iowa City, IA, United States; ^4^Department of Pediatrics and Interdisciplinary Genetics Program, University of Iowa, Iowa City, IA, United States

**Keywords:** X-linked Retinoschisis, retinoschisin, disease mechanisms, Caspase-1, immunology, apoptosis, electroretinogram

## Abstract

**Purpose:**

Previous studies have reported Caspase-1 (*Casp1*) is upregulated in mouse models of Juvenile X-linked Retinoschisis (XLRS), however no functional role for *Casp1* in disease progression has been identified. We performed electroretinogram (ERG) and standardized optical coherence tomography (OCT) in mice deficient in the Retinoschisin-1 (*Rs1*) and *Casp1* and Caspase-11 *(Casp11)* genes (*Rs1*-KO*;Casp1/11^−/−^*) to test the hypothesis that *Casp1* may play a role in disease evolution and or severity of disease. Currently, no studies have ventured to investigate the longer-term effects of *Casp1* on phenotypic severity and disease progression over time in XLRS, and specifically the effect on electroretinogram.

**Methods:**

*Rs1*-KO;*Casp1/11^−/−^* mice were generated by breeding *Rs1*-KO mice with *Casp1/11^−/−^* mice. OCT imaging was analyzed at 2-, 4-, and 15–16 months of age. Outer nuclear layer (ONL) thickness and adapted standardized cyst severity score were measured and averaged from 4 locations 500 μm from the optic nerve. Adapted standardized cyst severity score was 1: absent cysts, 2: <30 μm, 3: 30–49 μm, 4: 50–69 μm, 5: 70–99 μm, 6: >99 μm. Electroretinograms (ERG) were recorded in dark-adapted and light-adapted conditions at 2 and 4 months. Results obtained from *Rs1*-KO and *Rs1*-KO;*Casp1/11^−/−^* eyes were compared with age matched WT control eyes at 2 months.

**Results:**

Intraretinal schisis was not observed on OCT in WT eyes, while schisis was apparent in most *Rs1*-KO and *Rs1*-KO;*Casp1/11^−/−^* eyes at 2 and 4 months of age. There was no difference in the cyst severity score from 2 to 4 months of age, or ONL thickness from 2 to 16 months of age between *Rs1*-KO and *Rs1*-KO;*Casp1/11^−/−^* eyes. ERG amplitudes were similarly reduced in *Rs1*-KO and *Rs1*-KO;*Casp1/11^−/−^* compared to WT controls at 2 months of age, and there was no difference between *Rs1-*KO and *Rs1*-KO;*Casp1/11^−/−^* eyes at 2 or 4 months of age, suggesting no impact on the electrical function of photoreceptors over time in the absence of *Casp1*.

**Conclusion:**

Although *Casp1* has been reported to be significantly upregulated in *Rs1-*KO mice, our preliminary data suggest that removing *Casp1/11* does not modulate photoreceptor electrical function or alter the trajectory of the retinal architecture over time.

## 1 Introduction

Juvenile X-linked Retinoschisis (XLRS), the leading cause of macular dystrophy in young male patients, is an X-linked recessive retinal disorder resulting from mutations in the Retinoschisin-1 gene (*RS1*). These mutations lead to the dysfunction or deficiency of its gene product, retinoschisin-1 (RS1). RS1, characterized by a conserved discoidin domain, engages with cellular membranes and extracellular proteins within the retina, serving as an essential scaffold and is pivotal in upholding the structural integrity of the various retinal layers (1, 2). Consequently, in individuals with XLRS, intercellular retinal adhesion is impaired, culminating in the formation of retinal schisis, primarily within the inner plexiform layer (1).

In human subjects with XLRS, in addition to the formation of schisis cavities, an early progressive degeneration of the photoreceptor cell layer results in thinning of the retina. Affected children present with low vision, stellate maculopathy on fundus exam, cystic retinopathy on optical coherence tomography (OCT) and eventually electronegative b-waves on full field electroretinogram (ERG) (3). The formation of schisis, photoreceptor loss, and an electronegative ERG are recapitulated in the RS1 mouse model (4).

Recently, investigation into the immunologic profile of patients with XLRS has gained significant attention since the immune system plays a significant role in the safety and efficacy of gene therapies targeting the ocular compartment. In a phase-I/II clinical trial, the intravitreal delivery of gene therapy vectors resulted in an increase in inflammatory blood cell types (5, 6). Moreover, the phenotypic variability observed in XLRS, including variance within families sharing the same mutation, raises the possibility of a disease severity modifier, such as the individual’s immunologic profile, influencing both the timing of presentation and the severity of symptoms.

In a previous investigation conducted by Gehrig et al. (7) elevated expressions of inflammatory caspases (Caspase-1, −11, −12) were observed using quantitative real-time polymerase chain reaction (qRT-PCR) and western blot analyses in a mouse model of XLRS. Notably, the expression levels of all inflammatory caspases reached their peak at postnatal day (P) 21. Among them, Caspase-1 (*Casp1*) exhibited the most substantial increase in expression (7). However, in the absence of *Casp1*, as determined through the development of a knockout mouse model, it was revealed that during the early stages of disease progression, the extent of photoreceptor apoptosis did not significantly differ in the quantification of apoptotic photoreceptor cell death between *Rs1*-KO mice and *Rs1*-KO;*Casp1/11*^−/−^ mice (7). Additionally, the absence of *Casp1* did not result in a variance in the degree of activation of mouse retinal microglia (7). Even so, given the substantial upregulation of this potent inflammatory mediator in the model, further exploration is imperative. Currently, no studies have investigated the longer-term effects of *Casp1* on phenotypic severity and disease progression over time in a mouse model of XLRS, and specifically no studies have used ERG to study the potential effect on photoreceptor functioning.

In this study, we examined the role of *Casp1*, located on the mouse genome at 9A1, and its inflammatory enzyme product also known as interleukin-1β converting enzyme (ICE) (8). Caspase-1 plays a pivotal role as a key activator in the formation of pro-inflammatory cytokines, including interleukin-18 (IL-18) and interleukin-1 beta (IL-1β), which not only exacerbate inflammatory responses but also potentiate programmed cell death pathways. The *Casp1^−/−^* mouse model used contains an incidental mutation in Caspase-11. Due to their proximity in the genome, this mouse model is also deficient in Caspase-11, as Caspase-1 and Caspase-11 are unable to be segregated during recombination due to the nature of their location on the genome (9).

We hypothesized that the knockout of the *Casp1* gene in *Rs1*-KO mice would lead to a phenotypic alteration in the electrical function of the retina, measurable through ERG, and have an impact on the development and severity of retinoschisis, observable on OCT. Interestingly, our experiments yielded no discernible differences in disease progression over time, suggesting a limited or insignificant role for *Casp1* in the progression to later stages of disease, despite its documented upregulation in mouse models.

## 2 Methods

### 2.1 Study design

The experiment was designed to compare the phenotypic characteristics in mice lacking the *Rs1* gene with mice deficient in both the *Rs1* and the *Casp1/11* genes. Results were obtained at 2, 4, and 15–16 months of age. At the 2-month time point, the results were compared with wild-type (WT) control mice to establish a baseline phenotype that represents the expected characteristics in the absence of any disease or genetic modifications.

### 2.2 Animal husbandry and ethics statement

This study was performed in strict accordance with the recommendations in the Guide for the Care and Use of Laboratory Animals of the National Institutes of Health and/or adhered to the Association for Research in Vision and Ophthalmology Statement for the Use of Animals in Ophthalmic and Vision Research. All the animals were handled according to the approved Institutional Animal Care and Use Committee (IACUC) protocol #4031421 of the University of Iowa. The *Rs1*-KO (C57Bl/6 J) mouse model was generously provided by Dr. Paul Sieving MD, PhD. This mouse model contains a deletion of exon 1 and a 1,630 bp fragment of intron 1 of the *Rs1* gene (4).

The *Casp1/11^−/−^* (B6N.129S2-Casp1^tm1flv^/J-JAX stock #016621) mouse model was received from the Jackson Laboratory (10). The embryonic stem cell line used to create the *Casp1* KO contains a naturally occurring deficiency in Caspase-11 (10). Due to the location of the two genes on the genome, they cannot be segregated during backcrossing. Therefore, throughout this paper we refer to this mouse line as *Casp1/11^−/−^. Rs1*-KO;*Casp1/11^−/−^* mice were generated by breeding and backcrossing *Casp1/11^−/−^* mice with *Rs1*-KO mice. The mice used in the study are on a C57BL/6 J background. Mice were screened for the rd8 mutation by PCR followed by sequencing.

Both affected male and affected female knockout mice were utilized in this study. They were housed under 50-lux cyclic lighting (12 h on:12 h off) condition with open access to food and water. Humane endpoints were strictly observed, and the methods of euthanasia included carbon dioxide inhalation followed by cervical dislocation.

### 2.3 Genotyping

Genotyping to identify knock out mice was done using primers and cycling conditions listed in Table 1. Genotyping was performed using *Taq* DNA polymerase (M0273 S, New England BioLabs) and the primers listed in Table 1 following the manufacturer’s instructions. Amplifications were performed on the ABI 7900 real-time polymerase chain reaction (PCR) instrument. The PCR products were measured by agarose gel electrophoresis (*E-gel Invitrogen by Thermo Fisher Scientific, Israel*).

**Table 1:** Primers for *Rs1-*KO and *Casp1/11^−/−^.*

Primers for *Rs1* genotyping		
Primers	Ratio (%)	Sequence (5′–3′)
*RS1* Promoter F2	33	TAGGGGCCCACATCTTCCAAC
PLA2	33	GTTCTTCGGACGCCTCGTCAACAC
RSWT2-R	33	GTGACAAAGAGCCACACAACAGTGACC
WT band, 516 bp; KO band, 300 bp.

Primers for *Casp1* genotyping		
Primers	Ratio (%)	Sequence (5′–3′)
*Casp1* WT F	33	GAGACATATAAGGGAGAAGGG
Common R	33	ATGGCACACCACAGATATCGG
*Casp1* KO F	33	TGCTAAAGCGCATGCTCCAGACTG

WT band, 500 bp; KO band, 300 bp.

Cycling conditions for *Rs1* genotyping are as follows; initial denaturing at 94°C for 5 min followed by 35 cycles of denaturing at 94°C for 30 s, annealing at 57°C for 30 s, and extension at 72°C for 30 s, with a final extension step at 72°C for 4 min. This produces a 516-base pair (bp) wild-type band and a 300 bp knockout band.

Cycling conditions for the *Casp1^−/−^* genotyping are as follows; initial denaturing at 94°C for 5 min, followed by 35 cycles of denaturing at 94°C for 30 s, annealing at 58°C for 60 s, and extension at 72°C for 60 s, with a final extension step at 72°C for 7 min. This produces a 500-base pair (bp) wild-type band and a 300 bp knockout band. See Table 1 for primer details.

### 2.4 Optical coherence tomography (OCT) image acquisition and processing

**Figure 1:**
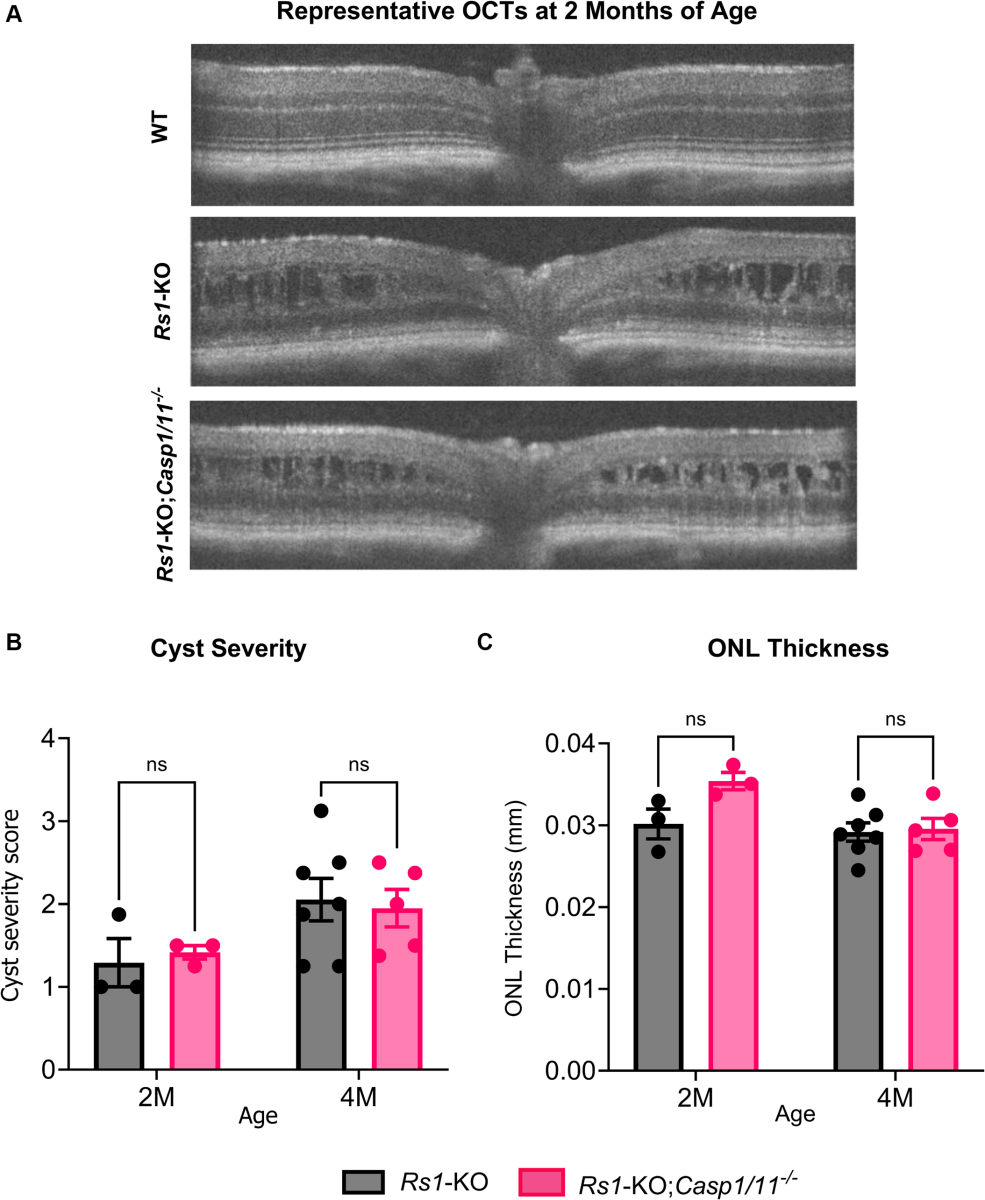
**(A–C)** Cyst severity and ONL thickness unaffected by the absence of *Casp1* in *Rs1*-KO eyes at 2–4 MO. OCT images of *Rs1*-KO and *Rs1*-KO;*Casp1/11^−/−^* were collected at 2 and 4 months of age. Representative OCT findings at 2 M **(A)**. Measurements of cyst severity were taken at four equidistant points 500 um from the optic nerve. For the purpose of OCT experiments, one eye is considered a biological data point. ONL thickness was also measured and quantified from these images **(C)**. For cyst severity **(B)**, cyst height was translated to a modified scoring scale of 1–6 and averaged for that eye. Cyst severity and ONL thickness (mm) were not significantly different between *Rs1*-KO and *Rs1*-KO;*Casp1/11^−/−^* at 2 or 4 months of age. These findings suggest that the absence of *Casp1* did not appear to influence the formation of cysts in *Rs1*-KO eyes or the loss of photoreceptor cell layer up to the age of 4 months.

Mice were anesthetized for OCT experiments using a ketamine/xylazine mixture (87.5 mg/kg ketamine, 12.5 mg/kg xylazine). Pupils were dilated with one drop of 1% tropicamide. To maintain corneal hydration, a 1% carboxymethylcellulose was applied during testing. High-definition spectral domain OCT images were obtained using the Envisu R2200 SD-OCT ophthalmic imaging system (Bioptigen, Durham, NC, USA). Volumetric scans were taken of the optic nerve along with lateral B scans on the nasal and temporal sides of the optic nerve. Axis manipulation before image capture was done to centrally align the optic nerve head (ONH) in horizontal scans. Cyst severity was scored using a standardized system adapted from previous literature (11). Cysts were measured and scored at 4 locations equidistant from the optic nerve head then averaged. Biotogen InvivoVue Clinic software was used for quantification. Adapted standardized cyst severity score was 1: absent cysts, 2: <30 μm, 3: 30–49 μm, 4: 50–69 μm, 5: 70–99 μm, 6: >99 μm (11). Data quantified from all 4 locations and both eyes for each mouse were averaged to represent a final value per mouse. At 2, 4, and 15–16 months of age, outer nuclear layer (ONL) thickness was measured from external limiting membrane (ELM) to outer margin of outer plexiform layer (OPL) (Figure 1). Cyst severity score was not calculated at the 15–16-month endpoint because all cysts have resolved before this timepoint. Mice were injected with 0.1 mg/mL of atipamezole to aid in recovery from the anesthetic.

### 2.5 Electroretinogram (ERG) recording and analysis

Mice were dark adapted overnight and anesthetized for ERG experiments using a ketamine/xylazine mixture (87.5 mg/kg ketamine, 12.5 mg/kg xylazine). Pupils were dilated with one drop of 1% tropicamide. To maintain corneal hydration during testing, 1% carboxymethylcellulose and 2.5% Hypromellose solution (Akron, Lake Forest, Illinois) were applied. ERG testing was performed using the Celeris system from Diagnosys (Lowell, MA). We followed a modified ISCEV protocol (12) to record full field ERG under dark and light adapted conditions. Electrodes were placed on the cornea of bilateral eyes, and impedance was maintained below 10 kΩ.

Dark adapted rod response was assessed in *Rs1*-KO and *Rs1*-KO;*Casp1/11^−/−^* eyes by subjecting eyes to 15 flashes of 0.01 cd•s/m^2^ light (0.01 dim flash), followed by 15 flashes of 3.0 cd•s/m^2^ light [3.0 standard combined response (SCR)]. Cone function was assessed by subjecting eyes to a 10-min light-adaptation followed by 15 flashes of 3.0 cd•s/m^2^ light (3.0 bright flash) and 20 flashes of a 5 Hz flickering light of 3.0 cd•s/m^2^ (5 Hz Flicker) against a light adapted background.

### 2.6 Statistical analysis

Analysis was performed using GraphPad Prism 10.0 (GraphPad Software, Inc., San Diego, California, USA). One-way ANOVA was used when analyzing the 2-month time point comparing the experimental cohorts to WT, followed by multiple comparisons (Tukey test). Two-way Anova (or mixed model) was used when analyzing ERG metrics at the 4-month time point, and was also used for all OCT metrics, followed by multiple comparisons (Sidak’s test). A Student t-test was used when analyzing the 15–16 month ONL thickness measurements. Statistical significance was determined by *α* = 0.05 and *p*-values are reported. Values are expressed as the mean ± standard deviation throughout the manuscript. *N* values are reported in-text and in Table 2.

**Table 2:** *N*-values *Rs1-*KO and *Rs1*-KO;*Casp1/11^−/−^* cohorts (eyes).

Primers	*Rs1*-KO	*Rs1*-KO;*Casp1/11^−/−^*
2 Months	7	3
4 Months	13	7
15–16 Months	6	10

## 3 Results

Cross breeding between *Rs1*-KO and *Casp1/11^−/−^* mice produced a mouse strain functionally deficient in both *Rs1* and *Casp1,* referred to in this study as *Rs1*-KO;*Casp1/11^−/−^*. This mouse strain is also deficient in Caspase-11, which was also knocked out in this study, as Caspase-1 and Caspase-11 are unable to be segregated during recombination due to the nature of their location on the genome (9). OCT and ERG were used to compare the functional outcomes between the two groups. OCTs were taken at 2–(2 M), 4–(4 M), and 15–16 (15–16 M) months of age. ERGs were collected at 2 M and 4 M. This timeline was selected to investigate longer-term outcomes in the *Rs1*-KO;*Casp1/11^−/−^* cohort, aiming to discern any notable effects of *Casp1* on disease progression compared to *Rs1*-KO mice, particularly regarding electrical function of the retina, photoreceptor layer thinning, and cyst severity. These findings could offer further insights into the mechanisms underlying functional photoreceptor cell loss in patients with XLRS.

### 3.1 Optical coherence tomography

A hallmark of XLRS is the development of intraretinal schisis and cysts. Previously, we have found that schisis in *Rs1-*KO mice appears as early as P15, remains severe in young adult mice between 2 to 4 months of age, and naturally resolves by approximately 6 months of age, accompanied by a progressive loss of the laminar structure of the retina (13). To investigate whether the deletion of the *Casp1* gene affected the retinal structures of *Rs1-*KO mice, OCT was performed at 2 M, 4 M, and 15–16 M of age in *Rs1-*KO and *Rs1-*KO*;Casp1/11^−/−^* mice. Notably, both *Rs1-*KO and *Rs1-*KO*;Casp1/11^−/−^* mice had evidence of schisis on OCT at 2 M and 4 M (refer to Figure 1).

To better determine whether there was a difference in the severity of schisis, the sizes of intraretinal cysts were quantified at 4 different positions in each eye and a severity score was calculated, with a score of 1 being the least severe (no cysts), and 6 being the most severe (cyst height > 99 μm) (11).

At 2 M, there was no significant difference in severity of cysts between *Rs1*-KO eyes (*n* = 7) and *Rs1-*KO*;Casp1/11^−/−^* (*n* = 3) eyes (2 M: *Rs1*-KO: 1.292 ± 0.50; *Rs1-*KO*;Casp1/11^−/−^*: 1.417 ± 0.14; *p* = 0.954) (Figure 1B). At 4 M, there continued to be no significant difference in cyst severity between *Rs1*-KO eyes (*n* = 13) and *Rs1-*KO*;Casp1/11^−/−^* (*n* = 7) eyes (4 M: *Rs1-*KO: 2.054 ± 0.68; *Rs1-*KO*;Casp1/11^−/−^*: 1.950 ± 0.51; *p* = 0.940) (Figure 1B). Therefore, the absence of *Casp1* does not alter formation or severity of intraretinal cysts in *Rs1-*KO mice.

XLRS phenotypical ONL thinning was observed in the *Rs1*-KO and *Rs1-*KO*;Casp1/11^−/−^* cohorts. At 2 M, ONL thickness (mm) in *Rs1*-KO eyes (*n* = 3) and *Rs1-*KO*;Casp1/11^−/−^* eyes (*n* = 3) was not significantly different from each other (2 M: *Rs1*-KO: 0.030 ± 0.003 mm; *Rs1-KO;Casp1/11^−/−^*: 0.035 ± 0.002 mm; *p* = 0.078) (Figure 1C). At 4 M, there continued to be no difference in ONL thinning between *Rs1*-KO (*n* = 7) and *Rs1-*KO*;Casp1/11^−/−^* (*n* = 5) eyes (4 M: *Rs1*-KO: 0.029 ± 0.003 mm; *Rs1-*KO*;Casp1/11^−/−^*: 0.030 ± 0.003 mm; *p* = 0.966), suggesting ONL thinning occurs regardless of a *Casp1* deletion and the function of *Casp1* does not hasten or worsen retinal photoreceptor cell layer thinning or conversely prevent retinal thinning (Figure 1C). Representative OCT findings at 2 M can be viewed in Figure 1A.

**Figure 2:**
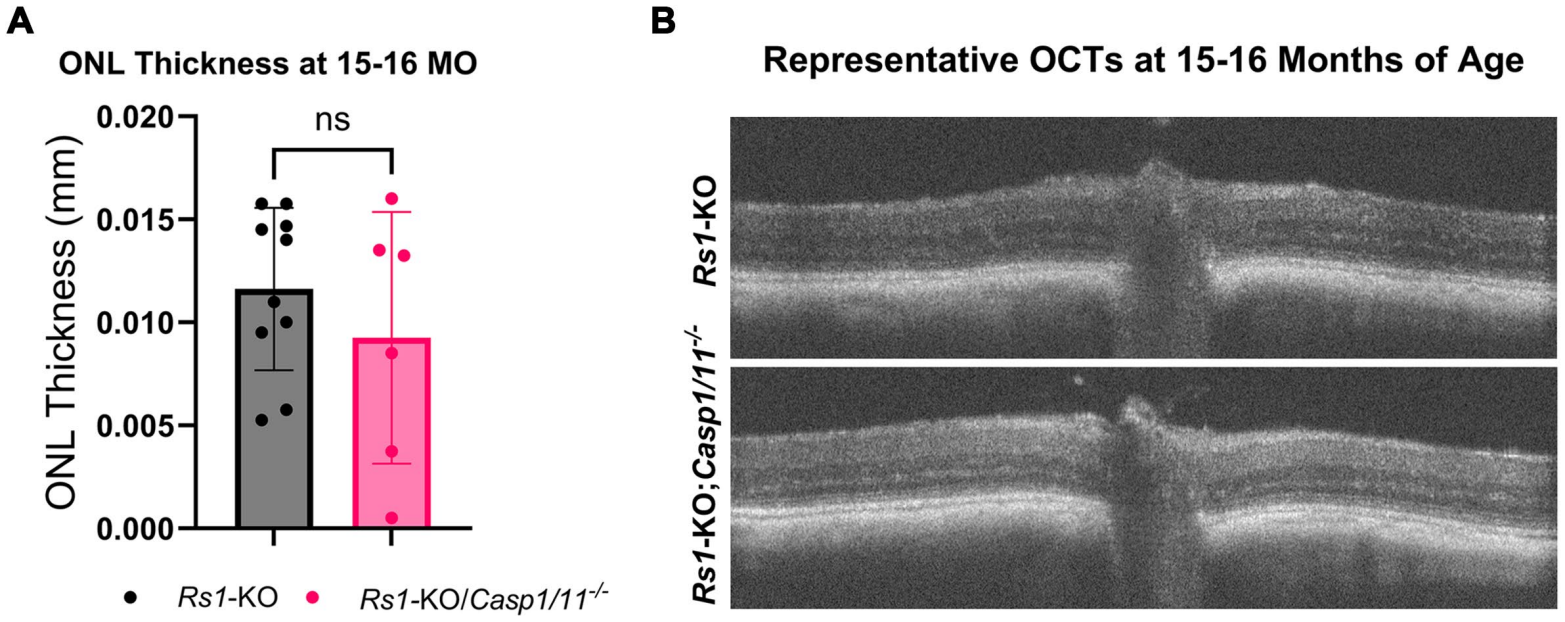
ONL thickness unaffected by the absence of *Casp1* in *Rs1*-KO eyes at 15–16 MO. OCT images of *Rs1*-KO and *Rs1*-KO;*Casp1/11^−/−^* eyes were collected at 15–16 months of age, and measurements of ONL thickness were taken. ONL thickness was not significantly different between *Rs1*-KO and *Rs1*-KO;*Casp1/11^−/−^* at 15–16 months of age, suggesting *Casp1* does not appear to influence long term ONL thinning in later stages of disease **(A)**. Representative OCT findings at 15–16 M **(B)**.

To accommodate a longer-term observation period consistent with late disease progression, ONL thickness was also measured in a separate cohort of mice at 15–16 M of age. From 4 M of age to 15–16 M of age, the thickness of the ONL in *Rs1-KO* mice decreased from 0.029 mm to 0.012 mm, showing that there continues to be a slow degeneration of photoreceptors during adulthood in this mouse model of XLRS. However, similar to earlier timepoints, there was no significance between *Rs1*-KO eyes (*n* = 10) and *Rs1-*KO*;Casp1/11^−/−^* eyes (*n* = 6) (15–16 M: *Rs1*-KO: 0.012 ± 0.004 mm; *Rs1-*KO*;Casp1/11^−/−^*: 0.009 ± 0.006 mm; *p* = 0.359) (Figure 2). Representative OCT findings at 15–16 M can be viewed in Figure 2B.

Overall, assessment of the retinas of *Rs1-KO* versus *Rs1-*KO*;Casp1/11^−/−^* mice showed that the elimination of *Casp1* does not alter the retinal structure and apparent disease course in a mouse model of XLRS.

### 3.2 Electroretinogram

#### 3.2.1 Dark-adapted

The retinal rod-dominant pathway was measured using two dark-adapted ERG assays; the dark-adapted 0.01 dim flash and the 3.0 SCR which is a combined rod-cone assay. ERGs were collected from *Rs1*-KO and *Rs1-*KO*;Casp1/11^−/−^* mice at 2 and 4 months of age (2 M; *Rs1*-KO *n* = 7; *Rs1*-KO;*Casp1/11^−/−^ n* = 3) (4 M; *Rs1*-KO n = 13; *Rs1-*KO*;Casp1/11^−/−^ n* = 7). Each datapoint represents the ERG value from a singular eye. At the 2-month age time point, a WT comparison was included to represent the expected phenotype that persists at all time points in WT mice (*n* = 3).

**Figure 3:**
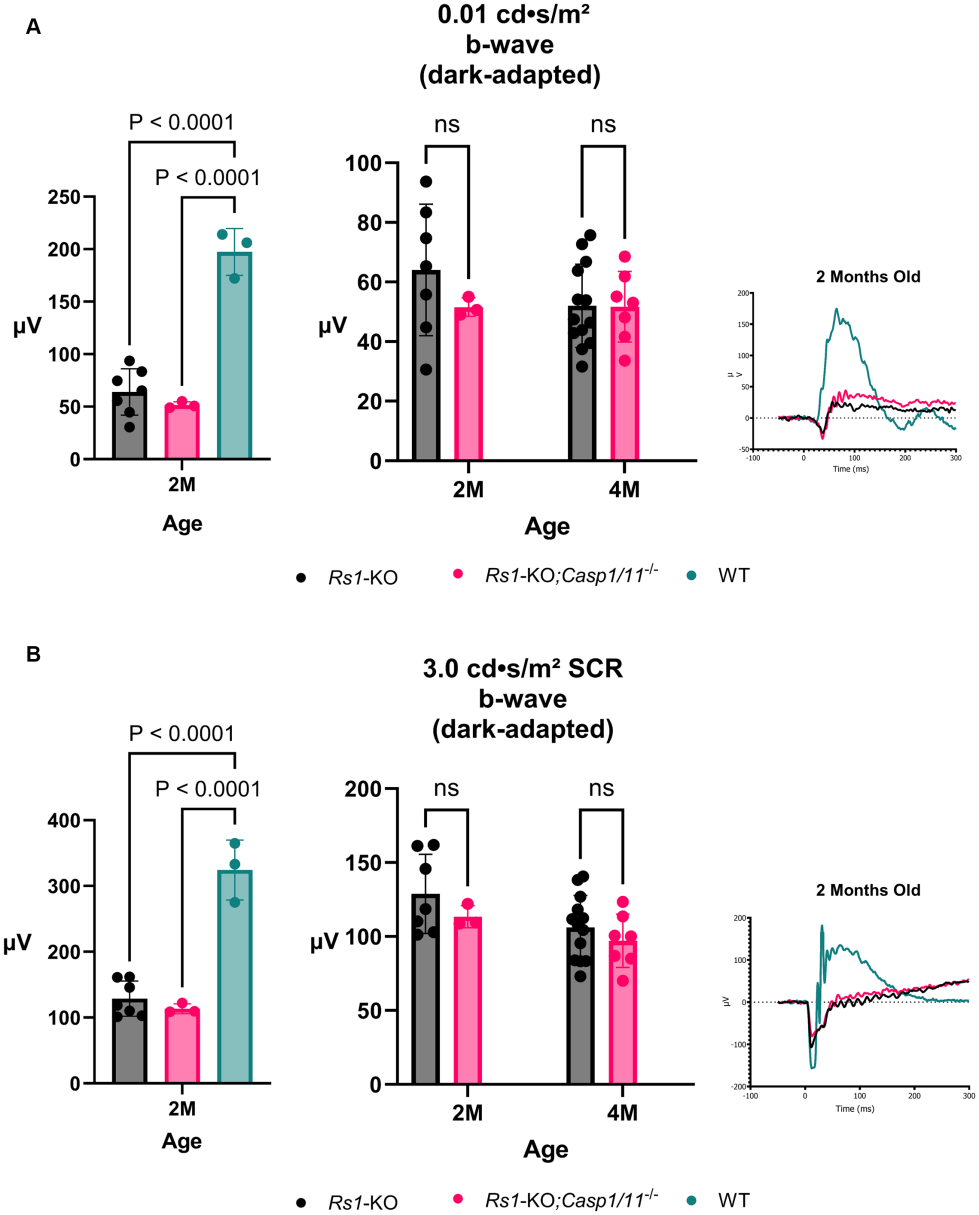
Rod-Pathway Function unaffected in the absence of *Casp1*. Rod-pathway function was assessed using dark-adapted ERG assays, including the 0.01 dim flash **(A)** and 3.0 SCR **(B)**, in *Rs1*-KO and *Rs1*-KO;*Casp1/11^−/−^* eyes at 2 and 4 months of age. For ERG experiments, one eye is considered a biological data point. At 2 M, *Rs1*-KO and *Rs1*-KO;*Casp1/11^−/−^* eyes were compared to wild-type (WT) eyes as controls. At 2 M, both experimental cohorts exhibited significantly reduced b-wave amplitudes when compared to the WT group (0.01 dim flash: *Rs1*-KO vs. WT: *p < 0.0001*; 3.0 SCR: *Rs1*-KO;*Casp1/11^−/−^* vs. WT: *p < 0.0001*). However, there was no significant difference between the *Rs1*-KO and *Rs1*-KO;*Casp1/11^−/−^* eyes, and this lack of difference persisted at the 4-month time point. This suggests that rod-pathway function may be minimally affected by the absence of the *Casp1* gene, even at the later stage of development. Representative ERG waveforms at 2 months of age visually demonstrate these findings **(A,B)**.

At 2 M, there was no significant differences found between the two cohorts in ERG values after the 0.01 dim flash stimulus (2 M: *Rs1*-KO: 64.034 ± 22.079 μV; *Rs1-*KO;*Casp1/11^−/−^*: 51.580 ± 3.118 μV; WT: 197.433 ± 22.31 μV; *p* = 0.445) or the 3.0 SCR (2 M: *Rs1*-KO: 128.841 ± 26.819 μV; *Rs1-*KO*;Casp1/11^−/−^*: 113.407 ± 7.45 μV; WT: 324.37 ± 45.45 μV; *p* = 0.508). As expected, both cohorts had significantly lower b-wave amplitudes than WT controls at 2 M in both tests (0.01 dim flash: *Rs1*-KO vs. WT: *p* < 0.0001; 3.0 SCR: *Rs1*-KO;*Casp1/11^−/−^* vs. WT: *p* < 0.0001), as demonstrated in Figure 3.

By 4 M, there continued to be no difference on ERG after the 0.1 dim flash stimulus (4 M: *Rs1*-KO: 52.016 ± 13.968 μV; *Rs1*-KO;*Casp1/11^−/−^*: 51.701 ± 11.829 μV; *p* = 0.998) or the 3.0 SCR stimulus (4 M: *Rs1*-KO: 106.117 ± 21.65 μV; *Rs1-*KO*;Casp1/11^−/−^*: 97.091 ± 18.07 μV; *p* = 0.608), demonstrated in Figure 3. These findings suggest that, without *Casp1*, there is no effect on the progressive function of rod-dominant electrical function of the retina and *Casp1* does not attenuate or modulate the rod response.

#### 3.2.2 Light-adapted

The retinal cone pathway was measured using two light-adapted ERG assays, the 3.0 bright flash and the 5 Hz Flicker. ERGs were collected from *Rs1*-KO and *Rs1-*KO*;Casp1/11^−/−^* mice at 2 and 4 months of age (2 M; *Rs1*-KO n = 7; *Rs1*-KO;*Casp1/11^−/−^ n* = 3) (4 M; *Rs1*-KO *n* = 13; *Rs1-*KO*;Casp1/11^−/−^ n* = 7). At the 2-month age time point, a WT comparison was included to represent the expected phenotype that persists at all time points in WT mice (*n* = 3).

**Figure 4:**
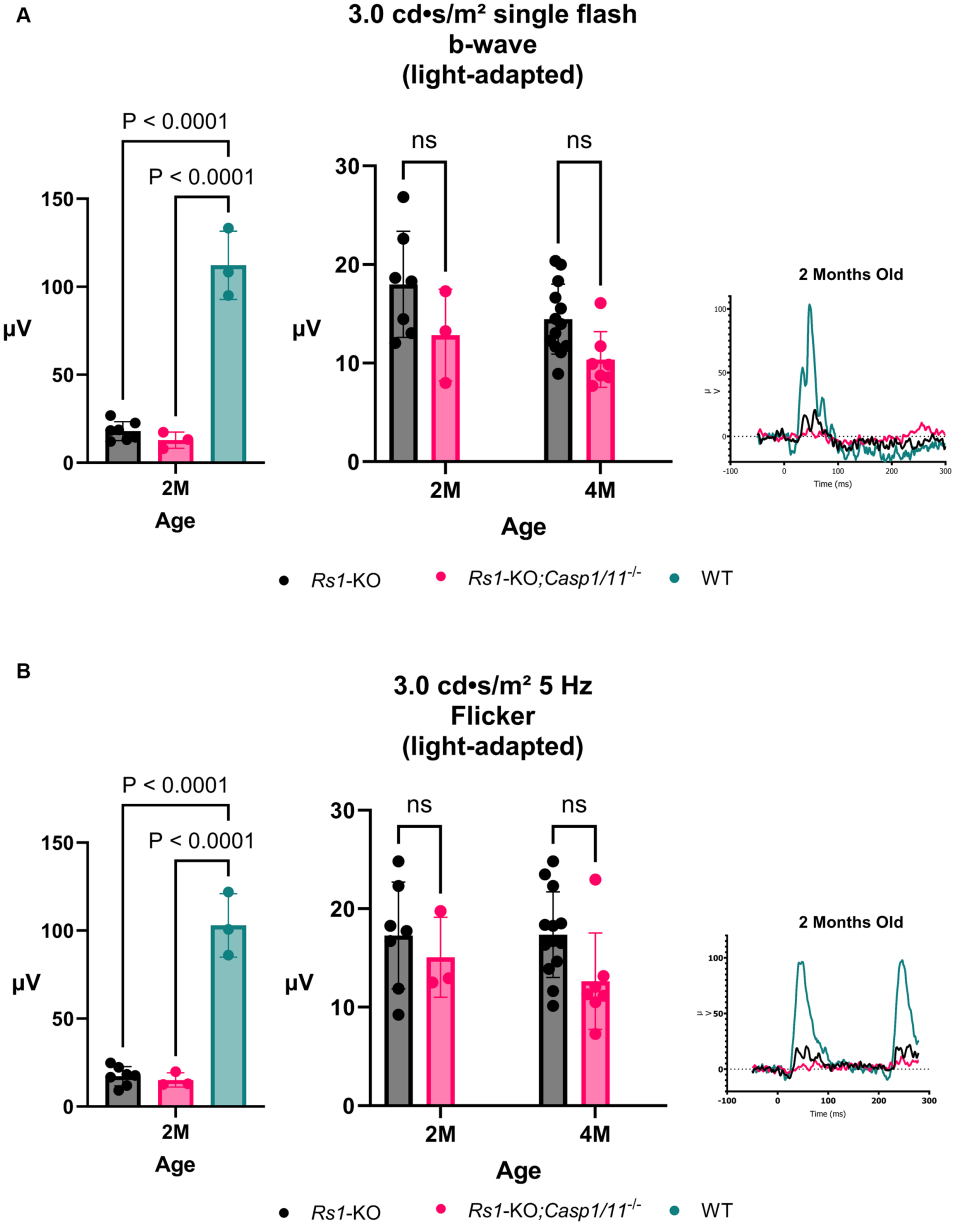
Cone-Pathway Function unaffected in the absence of *Casp1*. Cone-pathway function was assessed using light-adapted ERG assays, including the 3.0 bright flash **(A)** and 5 Hz Flicker **(B)**, in *Rs1*-KO and *Rs1*-KO;*Casp1/11^−/−^* eyes at 2 and 4 months of age. For the purpose of ERG experiments, one eye is considered a biological data point. At 2 M, *Rs1*-KO and *Rs1*-KO;*Casp1/11^−/−^* eyes were compared to wild-type (WT) eyes as controls. At 2 M, both experimental cohorts exhibited significantly reduced b-wave amplitudes when compared to the WT group (3.0 bright flash: *Rs1*-KO vs. WT: *p < 0.0001*; 5 Hz flicker: *Rs1*-KO;*Casp1/11^−/−^* vs. WT: *p < 0.0001*). However, there was no observable significant difference between the experimental cohorts in either light adapted measuring assay, at 2 or 4 months. This suggests that the *Casp1* gene may not affect cone pathway function at this later stage of development. Representative ERG waveforms at 2 months of age, visually demonstrate these findings **(A,B)**.

At 2 M, there was no significant difference between the cohorts found on ERG after the 3.0 bright flash stimulus (2 M: *Rs1*-KO: 17.986 ± 5.37 μV; *Rs1-*KO*;Casp1/11^−/−^*: 12.835 ± 4.66 μV; WT: 112.237 ± 19.37 μV; *p* = 0.1407) or the 5 Hz flicker stimulus (2 M: *Rs1*-KO: 17.283 ± 5.43 μV; *Rs1-*KO*;Casp1/11^−/−^*: 15.073 ± 4.07 μV; WT: 102.912 ± 18.02 μV; *p* = 0.754), demonstrated in Figure 4. Similar to the rod-measuring results, both cohorts had significantly lower b-wave amplitudes than WT controls in cone response (3.0 bright flash: *Rs1*-KO vs. WT: *p* < 0.0001; 5 Hz flicker: *Rs1*-KO;*Casp1/11^−/−^* vs. WT: *p* < 0.0001).

By 4 M, there continued to be no difference found in ERG values after the 3.0 bright flash stimulus (4 M: *Rs1*-KO: 14.461 ± 3.54 μV; *Rs1-*KO*;Casp1/11^−/−^*: 10.363 ± 2.84 μV; *p* = 0.0742) or the 5 Hz flicker stimulus (4 M: *Rs1*-KO: 17.355 ± 4.35 μV; *Rs1-*KO*;Casp1/11^−/−^*:12.638 ± 4.90 μV; *p* = 0.084), demonstrated in Figure 4. The absence of a cone effect once again suggests that *Casp1* may not have a role in certain long-term disease outcomes, particularly in electrical functioning of retinal cells.

## 4 Discussion

Previous animal studies consistently show an increase in Caspase-1 expression during retinal degeneration, whether it occurs through inheritance, as observed in a mouse model of Autosomal Dominant Retinitis Pigmentosa (ADRP) or induced through light-induced photoreceptor degeneration (14–16). However, when the downstream products of Caspase-1, IL-1β and IL-18, are blocked, retinal degeneration persists, suggesting that Caspase-1 itself is related to retinal degeneration, rather than its downstream effectors (14). This raises questions regarding the function of Caspase-1 in the setting of increased expression during retinal degeneration. Instead, Caspase-1 may have some other, yet unclear, function (14).

In their study, Gehrig et al. investigated the impact of a Casp1^−/−^ deletion on retinal cell apoptosis in both *Rs1-/Y* and *Rs1-/Y* mice (7). Utilizing TUNEL nuclei counts, they found no significant difference in the rate of apoptosis between the two groups. Additionally, through immunolabeling techniques, Gehrig et al. observed a notable decrease in resident microglia counts within the inner and outer retina of mice with the *Casp1* deletion, indicating a potential role of *Casp1* in regulating resident microglial populations (7). However, this increase in resident microglia was not accompanied by a corresponding change in the number of activated microglia, when *Rs1* was intact, suggesting that the presence of resident microglia is influenced by *Casp1*; however, the activation is independent (7).

The objective of this study was to determine if *Casp1* upregulation is the cause or consequence of the Rs1 phenotype, and further explore the potential factors contributing to its upregulation in mouse models (7). In ERG studies, no significant differences were found between *Rs1*-KO and *Rs1*-KO;*Casp1/11^−/−^* mice at 2 and 4 months of age. These results suggest that the absence of *Casp1* does not alter photoreceptor function.

Our findings revealed no significant differences in cyst severity between *Rs1*-KO mice and *Rs1*-KO;*Casp1/11^−/−^* mice, up to 4 months of age. Mice were followed to 15–16 months of age to investigate whether any effects on retinal morphology would emerge at later time points. However, our findings consistently showed no significant difference in ONL thickness between cohorts. Furthermore, it is noteworthy that early ONL thinning was observed irrespective of the absence of *Casp1*, indicating that the function of *Casp1* does not contribute to early or later-term outer nuclear layer retinal thinning, and does not have a measurable effect on total photoreceptor loss out to 16 months of age.

The immunological profile of human patients with XLRS continues to be a subject of great interest. Previous studies have reported differences in both innate and adaptive immune responses in XLRS human patients, including elevated baseline CD4/CD8 T-cell ratios and an increase in CD123+ and CD11c- plasmacytoid dendritic cells (DCs) (5). Notably, the ERK pathway, one of the four MAP Kinase pathways responsible for initiating cellular apoptosis, is upregulated in the early stages of XLRS pathogenesis (7, 17). In contrast, when retinoschisin is unimpaired and functional, there is a measurable downregulation of pro-apoptotic BAX proteins and Caspase-3, markers of apoptosis (17). Considering these finding, *Plössl* et al. proposed that the retinoschisin protein (RS1) is a potent regulator of MAP Kinase, and in the absence of functional RS1, MAP kinase potentiates apoptotic cell death (17). Their study underscores the protective role of RS1 against the apoptosis of retinal cells. Human patients also demonstrate elevated levels of IFN-γ and TNF-α when compared to healthy controls, suggesting an upregulated proinflammatory Th1 driven state (5). Interestingly, an upregulated Th1 response has been shown to induce tissue damage in other ocular sites like the cornea (18).

To understand how *Casp1* may influence the progression of disease in XLRS, it is important to grasp its more traditionally understood pathway. Associated with pyroptosis, a type of programmed cell death triggered by the presence of Pathogen-Associated Molecular Patterns (PAMPs) or Damage-Associated Molecular Patterns (DAMPs), Caspase-1 is a powerful activator in the setting of infection or tissue damage (19). The initiation of pyroptosis requires formation of a multiprotein inflammasome, assembled in response to DAMP recognition by pattern recognition receptors (PRR) (20). Formation of the inflammasome activates inflammatory caspase enzymes, including Casp1. These inflammatory caspases aid in the maturation and activation of proinflammatory cytokines through proteolytic enzyme activity (20). The activation of these cytokines, such as IL-1β and IL-18, induce formation of cellular membrane pores and ultimately cell rupture and death.

The traditional apoptotic function of CASP1 in humans has been well established in other tissues, including neuronal cell death in ischemic-reperfusion states (21) and the recruitment of immune cells in blood–brain barrier (BBB) injury (22). In mouse models lacking *Casp1*, there is a complete inability to convert pro-IL1β and pro-IL18 into mature proinflammatory cytokines (23). However, even though Casp1 is necessary for activation, studies involving the knockout of IL-18 in a mouse model of ADRP showed that retinal degeneration progressed similarly to controls, suggesting the proinflammatory products of Caspase-1 activation are not driving retinal degeneration (14). Similarly, our study demonstrates that complete *Casp1* knockout in the mouse model of XLRS still resulted in schisis formation, thinning of the ONL, and photoreceptor dysfunction, despite the assumed absence of proinflammatory cytokines, indicating IL-1β and IL-18 are unlikely mediators of photoreceptor loss and disease progression in this XLRS model.

Alternative roles of Caspase-1 outside the eye are currently under investigation, with studies indicating its presence and activation in non-immune cells even in the absence of IL-18 and IL-1β production (21). Caspase-1 also regulates the secretion of various proteins involved in the inflammatory process and tissue repair, suggesting a potential role in restoring homeostasis after significant biologic stress (22). It’s noteworthy that Caspase-1 also plays a role in metabolic processes, including the regulation of glucose balance, body weight, and lipid metabolism (21, 24). In human and mouse studies, Caspase-1 has a protective function against oxidative stress and injury in hepatocytes during period of ischemia and hemorrhagic shock (25). These alternative regulatory or protective roles provide valuable insights into the potential function of *Casp1* in response to cellular stress and debris, particularly in the context of retinal degeneration, as observed in XLRS.

In addition to immune system factors, various other elements contribute to disease severity. Genetic markers, immune system dynamics, and environmental factors collectively shape the course of XLRS and other diseases. While this research study has primarily centered on unraveling the impact of presence or absence of Caspase-1/11 on disease progression, it is vital to recognize the significance of environmental influences. Recent evidence suggests that alterations in fluid transport across the retina and diurnal rhythm play a role in the formation and timing of cyst severity and photoreceptor functioning (13, 26–28). Understanding the intricate interplay between these factors and immune system activation will be increasingly crucial for advancing our comprehension of XLRS and similar conditions.

One limitation of our study is the small sample size, which may impact the generalizability of our findings. A larger sample size is necessary to validate and further explain the results obtained in this study. Additionally, larger studies focusing on alternative functions of *Casp1* in various settings of retinal degeneration are warranted to investigate its role more comprehensively in XLRS and other retinal degenerative diseases.

While the precise mechanisms underlying XLRS and the potential involvement of *Casp1* remains elusive, our research provides valuable insights suggesting that *Casp1* is unlikely to be a driving force behind the retinal anatomic pathology and functional deficits associated with XLRS. The results of our study, when considered alongside the findings of Gehrig et al., which identified no discernible role for *Casp1* in influencing early retinal morphology or retinal immune cell activation, collectively suggests that *Casp1* does not have a significant impact on retinal function, and is unlikely to play a substantial role in modulating the primary structural or functional aspects of the XLRS mouse retina model (7). Understanding the limited role of *Casp1* in the pathogenesis of mouse XLRS further advances our knowledge of this condition in human subjects and the role the immune system plays in progressing disease, which can help guide the development of more treatments for affected individuals.

## Data availability statement

The original contributions presented in the study are included in the article/supplementary material, further inquiries can be directed to the corresponding author.

## Ethics statement

The animal study was approved by Institutional Animal Care and Use Committee (IACUC) protocol #4031421 of the University of Iowa. The study was conducted in accordance with the local legislation and institutional requirements.

## Author contributions

EG: Conceptualization, Data curation, Formal analysis, Investigation, Methodology, Project administration, Software, Supervision, Validation, Writing – original draft, Writing – review & editing. AP: Data curation, Software, Writing – original draft. JT: Conceptualization, Data curation, Formal analysis, Investigation, Methodology, Writing – review & editing. SB: Conceptualization, Writing – review & editing. PG: Conceptualization, Writing – review & editing. YH: Conceptualization, Formal analysis, Investigation, Methodology, Supervision, Writing – review & editing. AD: Conceptualization, Formal analysis, Funding acquisition, Investigation, Methodology, Project administration, Resources, Software, Supervision, Validation, Visualization, Writing – review & editing.
